# University of Ottawa constant load and speed rolling-element bearing vibration and acoustic fault signature datasets

**DOI:** 10.1016/j.dib.2023.109327

**Published:** 2023-06-18

**Authors:** Mert Sehri, Patrick Dumond, Michel Bouchard

**Affiliations:** a Department of Mechanical Engineering, University of Ottawa, 161 Louis Pasteur, Ottawa, Ontario, Canada; b General Bearing Service Inc., 490 Kent Street, Ottawa, Ontario, Canada

**Keywords:** Vibration, Machine condition monitoring, Fault detection/Diagnosis, Signal processing

## Abstract

The collection and analysis of data play a critical role in detecting and diagnosing faults in bearings. However, the availability of large open-access rolling-element bearing datasets for fault diagnosis is limited. To overcome this challenge, the University of Ottawa Rolling-element Bearing Vibration and Acoustic Fault Signature Datasets Operating under Constant Load and Speed Conditions are introduced to provide supplementary data that can be combined or merged with existing bearing datasets to increase the amount of data available to researchers. This data utilizes various sensors such as an accelerometer, a microphone, a load cell, a hall effect sensor, and thermocouples to gather quality data on bearing health. By incorporating vibration and acoustic signals, the datasets enable both traditional and machine learning-based approaches for rolling-element bearing fault diagnosis. Furthermore, this dataset offers valuable insights into the accelerated deterioration of bearing life under constant loads, making it an invaluable resource for research in this domain. Ultimately, these datasets deliver high quality data for the detection and diagnosis of faults in rolling-element bearings, thereby holding significant implications for machinery operation and maintenance.

**Specifications Table**

**Table:** 

Subject	Mechanical Engineering
Specific subject area	Vibration, machine condition monitoring, fault detection and diagnosis
Type of data	*.mat* (MATLAB file), *.xlsx* (Excel file), .*csv* (comma-separated values file) and *.png* (portable network graphics file)
How the data were acquired	An accelerometer, microphone, load cell, hall effect sensor, and two thermocouples were used to collect vibration, acoustic, load, rotational speed, and temperature data, respectively.
Data format	Raw, Processed
Description of data collection	Bearings are run from healthy to failure. This dataset includes ball, outer race, inner race, and cage faults. In addition, bearing health condition data under constant loads and speeds are collected. A total of 420,000 samples are collected at a sampling rate of 42,000 samples per second.
Data source location	Institution: University of Ottawa City/Town/Region: Ottawa, Ontario Country: Canada
Data accessibility	**Repository name:** Mendeley Data **Data identification number:** **Raw & Processed Data doi:**10.17632/y2px5tg92h.2 **Direct URL to data:** Raw, Processed Data: https://data.mendeley.com/datasets/y2px5tg92h

## Value of the Data

• The data can be used with different rolling-element bearing fault diagnosis and identification methods.
• The data collected can be used by machine learning researchers to train machine learning algorithms for rolling-element bearing fault diagnosis, either in the raw format, or after being processed (spectrograms) by combining and enhancing other publicly available datasets (e.g. CWRU [1], and PADERBORN [2]).
• Spectrograms can be used to train convolutional neural networks (CNNs) to perform rolling-element bearing diagnosis.
• Using data from other existing datasets, in combination with this dataset encourages the development of transfer learning and generalization.

## 1 Objective

Signals from an accelerometer, a microphone, a load cell, a hall effect sensor, and two thermocouples obtained from a machine operating under steady loads and speeds are provided in the dataset. This dataset offers a platform for evaluating the efficacy of traditional and machine learning-based fault identification methods in stable environments. Although this dataset is not large or diverse enough on its own to conduct deep learning analysis, it can be combined with other publicly available datasets such as CWRU [1], and PADERBORN [2]. Using this data, different approaches, methods, and algorithms can be evaluated to see how well they can identify and diagnose bearing faults. The dataset, combined with other datasets, also has the potential to help in training deep learning algorithms. Researchers can use this data, along with other available datasets, to improve the precision and dependability of machine-learning techniques, as well as the development of robust transfer learning strategies and generalization [3].

## 2 Data Description

### 2.1 Raw data description

Each data sample measures 10 s in length and is collected at a sampling rate of 42,000 Hz. The first column provides accelerometer data, the second column is acoustic data, the third column is motor speed, the fourth column is the load, and the fifth column is the temperature data. The raw data is provided as time series vibration and acoustic amplitudes, whereas motor speeds, applied load, and temperature are provided as is. Each raw data sample is labeled with the format {Letter}-{Number}-{Number}.

The letter in the dataset numbering indicates the final condition of the bearing being tested. Specifically, “H” denotes a healthy bearing, “I” indicates an inner race fault, “O” represents an outer race fault, “B” signifies a ball fault, and “C” indicates a cage fault.

The first number in the dataset numbering identifies the specific bearing that was tested. The second number indicates the condition of the bearing's health, where “0” indicates a healthy bearing, “1” signifies a developing fault, and “2” denotes a faulty bearing.

For example, the data sample labeled “I-2-1” corresponds to an inner race fault in bearing 2 with a developing fault, whereas “I-2-2” represents an inner race fault in bearing 2 that is faulty. Similarly, “O-6-1” stands for an outer race fault in bearing 6 with a developing fault, while “O-7- 2” indicates an outer race fault in bearing 7 that is faulty.

Table 1 provides a detailed breakdown of the dataset numbering scheme.

**Table 1:** Dataset numbering.

Bearing health condition	Constant load and speed 0 N, 400 N and 1750 RPM				
Healthy	H-1-0	H-2-0	H-3-0	H-4-0	H-5-0
	H-6-0	H-7-0	H-8-0	H-9-0	H-10-0
	H-11-0	H-12-0	H-13-0	H-14-0	H-15-0
	H-16-0	H-17-0	H-18-0	H-19-0	H-20-0
Developing fault (inner race)	I-1-1	I-2-1	I-3-1	I-4-1	I-5-1
Faulty (inner race)	I-1-2	I-2-2	I-3-2	I-4-2	I-5-2
Developing fault (outer race)	O-6-1	O-7-1	O-8-1	O-9-1	O-10-1
Faulty (outer race)	O-6-2	O-7-2	O-8-2	O-9-2	O-10-2
Developing fault (ball)	B-11-1	B-12-1	B-13-1	B-14-1	B-15-1
Faulty (ball)	B-11-2	B-12-1	B-13-2	B-14-2	B-15-2
Developing fault (cage)	C-16-1	C-17-1	C-18-1	C-19-1	C-20-1
Faulty (cage)	C-16-2	C-17-2	C-18-2	C-19-2	C-20-2

**Fig. 1:**
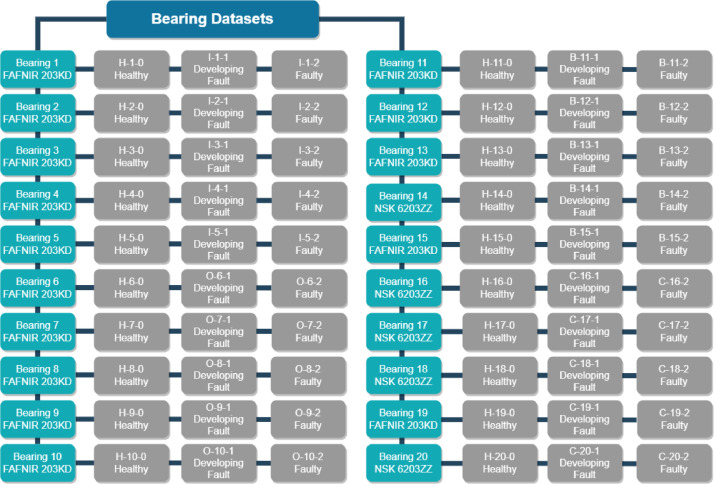
Dataset design block diagram.

Fig. 1. provides the block diagram for the dataset design, including each bearing state and manufacturer.

**Fig. 2:**
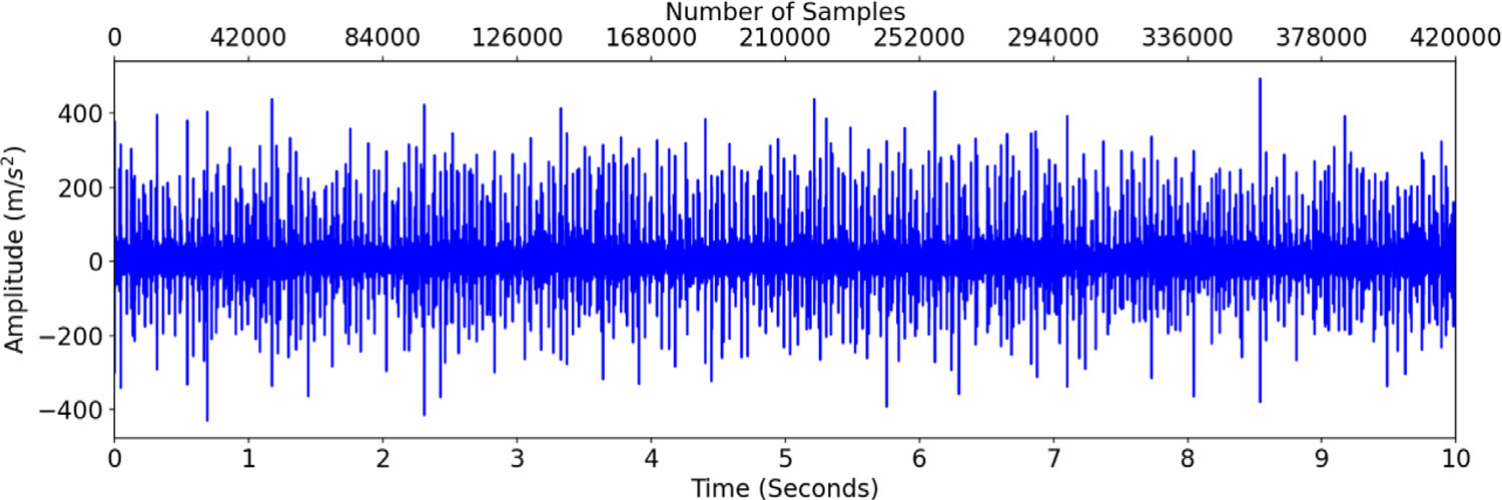
Accelerometer data for O-7-2.

**Fig. 3:**
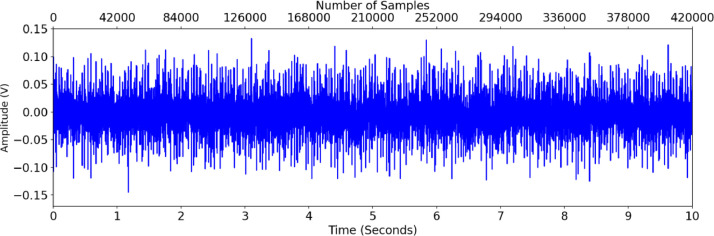
Acoustic data for O-7-2.

**Fig. 4:**
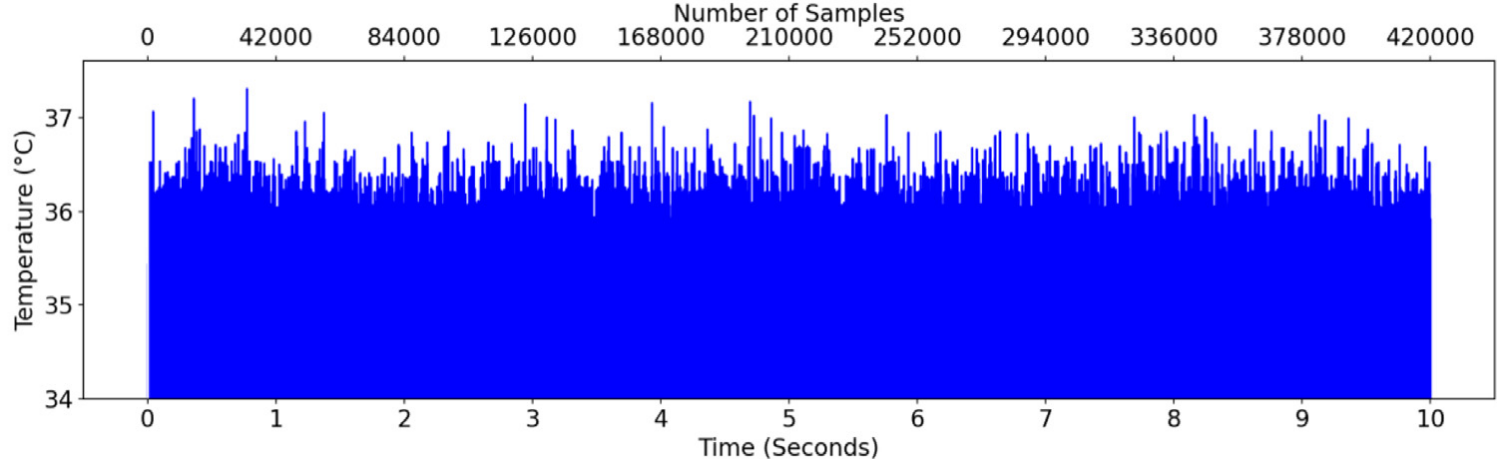
Temperature differential data for O-7-2.

**Fig. 5:**
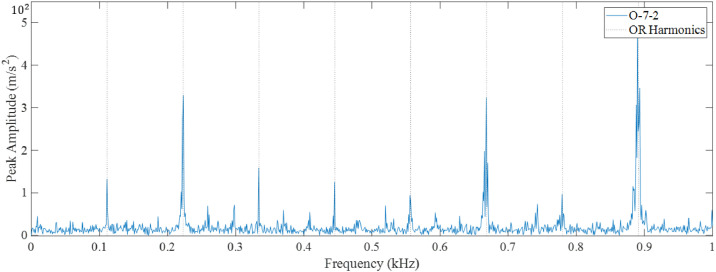
FFT Validation for O-7-2.

A sample of O-7-2 (bearing 7 outer race fault) is shown in Fig. 2 (vibration data), Fig. 3 (acoustic data), and Fig. 4 (temperature differential data). Fig. 5 provides the fast Fourier transform (FFT) for the same accelerometer sample, showing a potential outer race fault.

### 2.2 Processed data description

The processed dataset provides spectrogram images created from the raw accelerometer and microphone data described in Section 2.1. The spectrograms were created by processing the raw data using a Hanning window and the short-time Fourier transform (STFT) method. These pre-processed spectrograms are made available to researchers that wish to apply image-based methods for fault detection or diagnosis, saving time in transforming the raw data. The image size is 512 in both the *x* and *y* directions. 90% overlap is used to generate high-resolution spectrograms. The *x*-axis represents the number of samples (512), while the *y*-axis represents the frequency in kHz (0.512 kHz). A total of 24,000 images are provided in each set of data (12,000 accelerometer-based images and 12,000 microphone-based images). 20 healthy sets of images are provided, and the remainder are developing fault and faulty. Each spectrogram is labeled with the format {Letter}-{Number}-{Number}-{Number}.

**Fig. 6:**
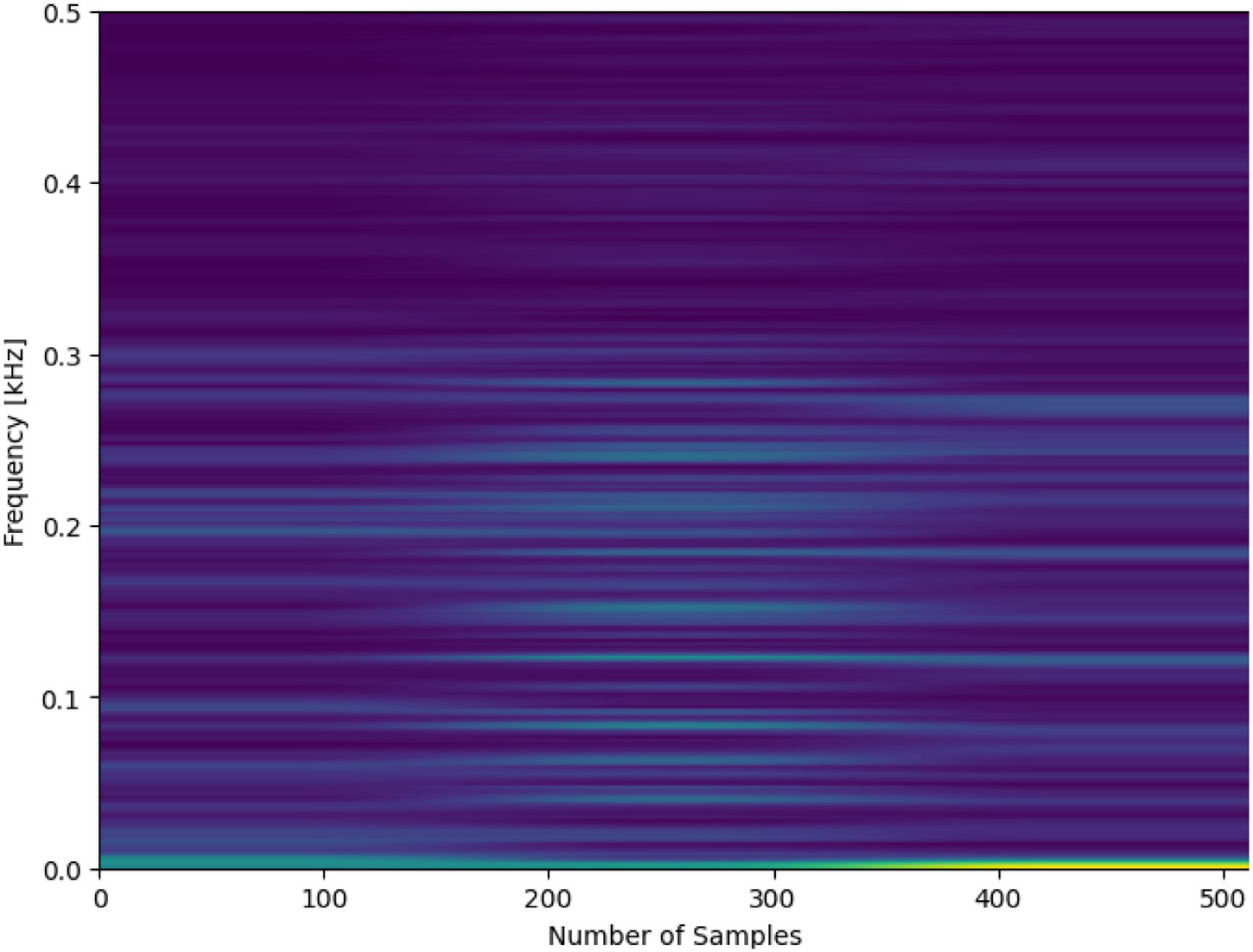
Spectrogram-processed acoustic data for C-17-2-2.

The spectrogram data follows the same format as the previous data, with an additional number included at the end that represents the spectrogram image number. For example, “I-2-1-0” represents the first spectrogram image of an inner race fault in bearing 2 with a developing fault, whereas “I-2-2-47” represents the 48^th^ spectrogram image of an inner race fault in bearing 2 that is faulty. Similarly, “O-6-1-399” indicates the last spectrogram image of an outer race fault in bearing 6 with a developing fault. A sample spectrogram is provided in Fig. 6.

## 3 Experimental Design, Materials and Methods

### 3.1 Dataset design

According to the audible range the maximum frequency of interest was selected as 20,000 Hz. The sampling rate (42,000 Hz) is chosen based on the data acquisition system and the Nyquist theorem [4]. Based on the requirements of many deep learning algorithms (i.e. the minimum limiting factor in this dataset design), a high number of samples are required. Therefore, using the defined sampling frequency, 10 s of data collection is deemed suitable as it provides a total of 420,000 data samples for each set of data. Each bearing has natural and stochastic inclusions that occur during material processing and component manufacturing that locally increase material stresses and lead to subsurface cracks [5]. Therefore, each set of data (i.e. 420,000 samples) provides a different result. Nonetheless, the data collection procedure can be replicated due to constant variables selected with respect to sampling rate (42,000 Hz), and the number of samples collected per sample (420,000). Moreover, the experimental test setup was designed to ensure that bearing failure occurred in a similar way each time (irrespective of the failure mode), and that data collection was consistent across all experiments. Lastly, the dataset was designed to contain 5 different sets of data for each type of fault (inner race, outer race, ball, cage), collected with a total of 20 rolling-element bearings.

Each set of bearing data is selected from a pool of 50 files collected over time, and each set of data was plotted with an FFT to ensure the samples had a clean signal that allowed for fault type validation. The faults were both identified visually and validated with the plotted FFTs. Three bearing conditions (healthy, developing fault, and faulty) are selected for data collection. Healthy data (selected within the first 5 files) is collected as a baseline for comparison with the developing fault data (selected within files 25 to 30) to see if a diagnosis can be made at the bearing's half-life point. Faulty data (selected within files 45 to 50) is collected for bearing fault diagnosis.

The bearing specifications used in these datasets are provided in Table 2.

**Table 2:** Bearing specifications.

Bearing model	Pitch diameter	Ball diameter	Number of balls
NSK 6203ZZ	28.50 mm	6.77 mm	8
FAFNIR 203KD	28.50 mm	6.77 mm	8

There are 60 sets of raw data and spectrograms converted from the raw data available at https://data.mendeley.com/datasets/y2px5tg92h [6].

### 3.2 Raw dataset design

The data for healthy bearings, as well as bearings with inner, outer, and cage faults, was recorded under a consistent nominal load of 400 N. For ball fault data, no load was applied because ball faults developed naturally within a reasonable amount of time under degreased conditions when compared to the other faulty types.

### 3.3 Processed dataset design

The spectrograms are processed by importing the accelerometer and acoustic data as a single column to Python. The code to create the spectrograms is provided as a GitHub link and provided with the data repository. A total of 60 sets of images are also included, processed from the 60 raw sets of data described in Section 3.1.1. The signal length used for creating the spectrograms is 512 samples and each dataset contains 400 images.

### 3.4 Experimental setup

**Fig. 7:**
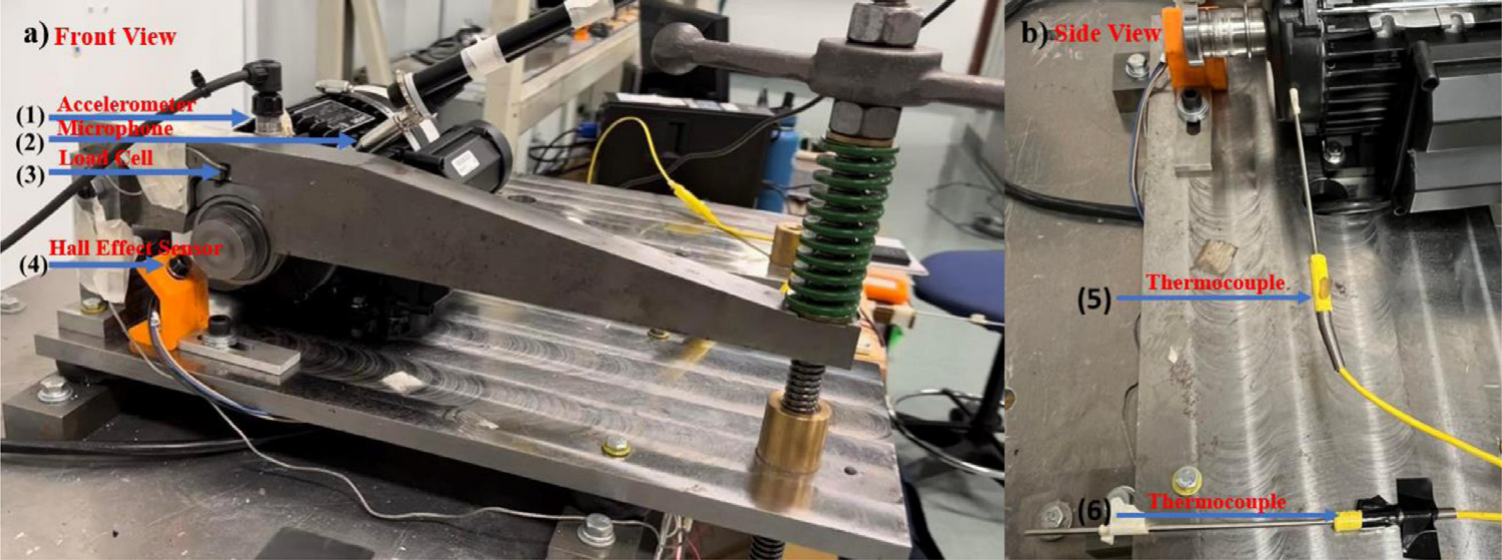
UORED-VAFCLS test rig set up in operation a) Front view b) Side view.

The data collection is performed using the University of Ottawa Rolling-element Dataset – Vibration and Acoustic Faults under Constant Load and Speed conditions (UORED-VAFCLS) test rig. The experimental setup is shown in Fig. 7. The experimental test rig consists of a single-phase motor mounted on a rigid plate supported by anti-vibration mounts. The motor shaft is stepped up using a shaft adapter, on which an SKF E22206 spherical roller bearing is mounted to withstand the load applied by the cantilever beam. The load applied by the beam is controlled via a lead screw. The motor is driven at a constant nominal speed of 1,750 RPM. The motor shaft is supported by two internal NSK 6203ZZ steel ball bearings. The drive-end bearing model got changed after 5 tests; the first 5 tests included the 6203ZZ bearings and the subsequent 15 tests consisted of FAFNIR 203KD bearings. The drive end bearing seals were removed and the bearings were degreased to accelerate deterioration during testing. They were then replaced after each test.

**Fig. 8:**
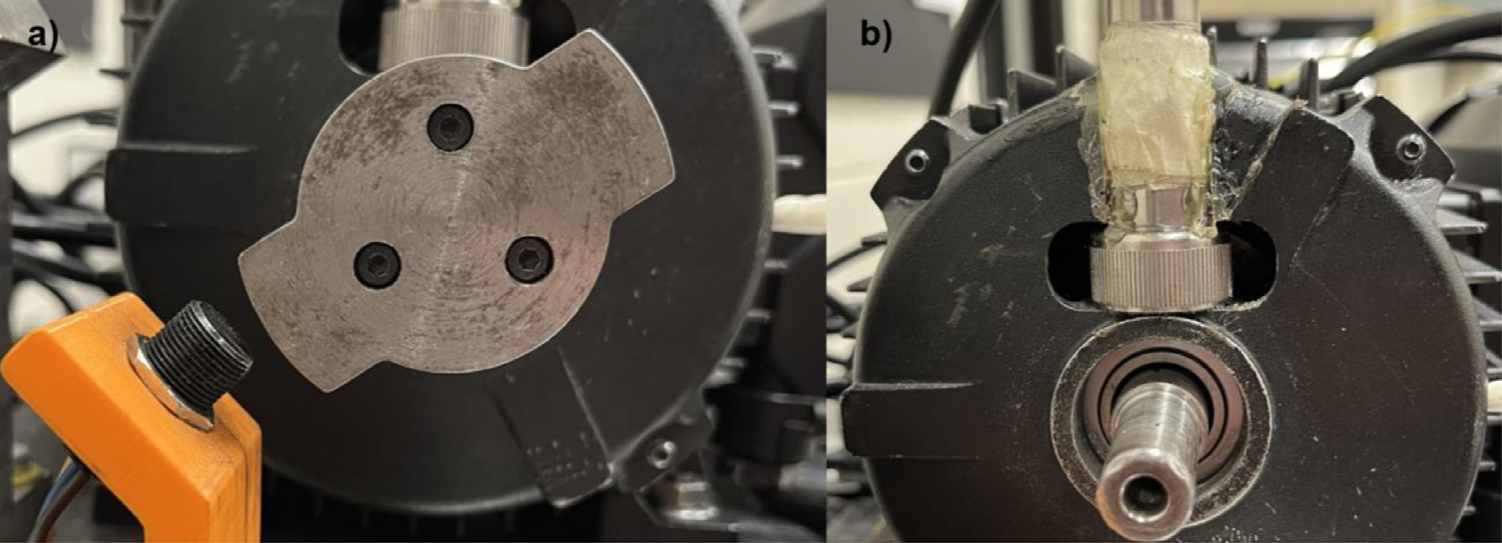
a) Two-toothed gear, b) Accelerometer placement (Front view – close-up).

The test rig shown in Fig. 7 has six sensors as follows: (1) accelerometer, (2) microphone, (3) load cell, (4) hall effect sensor, (5) motor temperature thermocouple, (6) room temperature thermocouple. The accelerometer (PCB, model 623C01) is mounted directly on the drive end bearing using a magnet by altering the motor casing, as shown in Fig. 8b. The microphone (PCB, model 130F20) is placed within 2 cm of the motor drive-end bearing being tested, but is supported independently of the motor. The load cell (OMEGA, model LCM302) is mounted between the cantilever arm, and the SKF E22206 bearing used to transfer the load to the motor shaft. The hall effect sensor (OMEGA, model OMDC-MPU-A), used to determine the motor's rotational speed, is mounted on an adjustable plastic support, and placed 2 mm away from the two-toothed gear. The two-toothed gear is mounted to the end of the drive shaft adapter, as seen in Fig. 8a. The thermocouples (OMEGA, model KTSS-HH) are used to determine the temperature differential of the bearing by collecting room temperature and bearing outer race temperature.

The experimental setup allows faults to be created in the motor drive end bearing and for the collection of data with a minimal signal-to-noise ratio. The accelerometer sensor placement was tested on both the outside and the inside of the motor casing (directly mounted on the bearing being tested itself). The accelerometer mounted inside the motor casing was found to have a significant noise reduction when compared to outside the casing, without suffering from electromagnetic field problems. All provided data was recorded when the accelerometer was mounted inside the motor casing. This will allow researchers to focus on analyzing a clean bearing signal with reduced noise.

### 3.5 Data acquisition system

A National Instruments USB-6212 data acquisition system is used to connect the sensors to the computer. The accelerometer and the microphone sensors are attached to a PCB Piezotronics 482C signal conditioner. The accelerometer is used to collect vibration signals, the microphone collects acoustic signals, the load cell collects load data, the hall effect sensor is used to collect the rotational speed of the motor shaft, and the thermocouple sensors are used to collect room temperature and bearing temperature.

### 3.6 Sampling frequency and length

The raw hall effect sensor data is converted to RPM by converting the voltage to frequency = 1 / (2 × period) × 60 (two gear teeth per rotation). The load cell, accelerometer, and thermocouple values are captured in V, the data is then converted to N, $\frac{m}{s^{2}}$, and (${}^{\circ }C$), respectively, using the sensitivity conversion rates provided by the manufacturer. The temperature differential is calculated by subtracting the room temperature from the bearing temperature. Therefore, the data presented in each raw data file have the following units: vibration ($\frac{m}{s^{2}}$), acoustic sound (V), load (N), rotational speed (RPM), and temperature (${}^{\circ }C$).

## Ethics Statement

The authors declare that they did not conduct human or animal studies.

## Credit Author Statement

**Mert Sehri:** Conceptualization, Methodology, Validation, Investigation, Data curation, Visualization, and Writing; **Patrick Dumond:** Reviewing, editing, and supervision; **Michel Bouchard:** Resources, Funding acquisition.

## Declaration of Competing Interest

The authors declare that they have no known competing financial interests or personal relationships that could have appeared to influence the work reported in this paper.

## Data Availability

University of Ottawa Rolling-element Dataset – Vibration and Acoustic Faults under Constant Load and Speed conditions (UORED-VAFCLS) (Original data) (Mendeley Data)

## References

[1] Download a Data File | Case School of Engineering | Case Western Reserve University (2021). https://engineering.case.edu/bearingdatacenter/download-data-file.

[2] “Konstruktions- und Antriebstechnik (KAt) - Data Sets and Download (Universität Paderborn) https://mb.uni-paderborn.de/kat/forschung/kat-datacenter/bearing-datacenter/data-sets-and-download (Accessed 19 May 2023).

[3] Zhang S., Zhang S., Wang B., Habetler T.G. (2020). Machine learning and deep learning algorithms for bearing fault diagnostics – a comprehensive review. IEEE Access.

[4] Kester W. (2023).

[5] Guan J., Wang L., Zhang C., Ma X. (2017). Effects of non-metallic inclusions on the crack propagation in bearing steel. Tribol. Int..

[6] Sehri M., Dumond P. (2023). University of Ottawa rolling-element dataset – vibration and acoustic faults under constant load and speed conditions (UORED-VAFCLS).

